# Identification of N7-methylguanosine related signature for prognosis and immunotherapy efficacy prediction in lung adenocarcinoma

**DOI:** 10.3389/fmed.2022.962972

**Published:** 2022-08-24

**Authors:** Zhouhua Li, Wenjun Wang, Juan Wu, Xiaoqun Ye

**Affiliations:** Department of Respiratory Diseases, The Second Affiliated Hospital of Nanchang University, Nanchang, China

**Keywords:** N7-methylguanosine, lung adenocarcinoma, molecular subtype, prognosis, immunotherapy efficacy

## Abstract

**Background:**

Lung adenocarcinoma (LUAD) is one of the most frequent causes of tumor-related mortality worldwide. Recently, the role of N7-methylguanosine (m^7^G) in tumors has begun to receive attention, but no investigation on the impact of m^7^G on LUAD. This study aims to elucidate the significance of m^7^G on the prognosis and immunotherapy in LUAD.

**Methods:**

Consensus clustering was employed to determine the molecular subtype according to m^7^G-related regulators extracted from The Cancer Genome Atlas (TCGA) database. Survival, clinicopathological features and tumor mutational burden (TMB) analysis were applied to research molecular characteristics of each subtype. Subsequently, “limma” package was used to screen differentially expressed genes (DEGs) between subtypes. In the TCGA train cohort (*n* = 245), a prognostic signature was established by univariate Cox regression, lasso regression and multivariate Cox regression analysis according to DEGs and survival analysis was employed to assess the prognosis. Then the prognostic value of the signature was verified by TCGA test cohort (*n* = 245), TCGA entire cohort (*n* = 490) and GSE31210 cohort (*n* = 226). Moreover, the association among immune infiltration, clinical features and the signature was investigated. The immune checkpoints, TMB and tumor immune dysfunction and exclusion (TIDE) were applied to predict the immunotherapy response.

**Results:**

Two novel molecular subtypes (C1 and C2) of LUAD were identified. Compared to C2 subtype, C1 subtype had poorer prognosis and higher TMB. Subsequently, the signature (called the “m^7^G score”) was constructed according to four key genes (*E2F7*, *FAM83A*, *PITX3*, and *HOXA13*). The distribution of m^7^G score were significantly different between two molecular subtypes. The patients with lower m^7^G score had better prognosis in TCGA train cohort and three verification cohort. The m^7^G score was intensively related to immune infiltration. Compared with the lower score, the higher m^7^G score was related to remarkable upregulation of the PD-1 and PD-L1, the higher TMB and the lower TIDE score.

**Conclusion:**

This study established a m^7^G-related signature for predicting prognosis and immunotherapy in LUAD, which may contribute to the development of new therapeutic strategies for LUAD.

## Introduction

Lung adenocarcinoma (LUAD) accounts for the largest proportion in non-small cell lung cancer (NSCLC) (1). Since patients with LUAD suffer from advanced disease or have distant metastasis when first diagnosed, they have a poor prognosis, and the overall 5-year survival rate is still below 20% (2, 3). Impressively, immune checkpoint blockade (ICB) has become a promising therapy strategy for NSCLC (4). However, some patients have a low response rate to ICB treatment, or even drug resistance, thus resulting in disease relapse or dead cases (5, 6). Therefore, it is essential to identify a novel biomarker in LUAD, in order to improve the outcomes of patients and formulate personalized treatment strategies.

Increasing evidence indicates that the initiation and progression of lung cancer depends not only on genetic variation, but also on epigenetic dysregulation (7, 8). As an important part of epigenetic modification, RNA modification is involved in regulating many physiological processes and disease occurrence (9). Besides, dynamic regulation and disruption of these RNA modifications are also related to the tumorigenesis, maintenance and progression of lung cancer (10, 11). Among numerous RNA dynamic modifications, N6-Methyladenosine (m^6^A), 5-Methylcytosine (m^5^C), and N7-methylguanosine (m^7^G) are extremely common (12). Importantly, m^7^G is the most prevalent modifications of RNA caps (13), which occurs in various RNAs of eukaryotes (14). m^7^G modification has a significant impact on RNA metabolism, processing and function (15). Nevertheless, the exploration of m^7^G-related regulators on tumors have only recently begun to receive attention owing to technological limitations. Mis-regulated m^7^G modification could disturb the translation of many oncogenic transcripts involved in RPTOR/ULK1/autophagy pathway, which contributed to esophageal squamous cell carcinoma oncogenesis (16). *EIF4E* is regarded as one of m7G-related regulators, whose phosphorylation could increase the translations of oncogene mRNAs to promote prostate cancer tumorigenesis (17). Moreover, one study demonstrated that *METTL1* and *WDR4* were upregulated in lung cancer samples and vital for the progression (18). Besides, RNA dynamic modification could influence the response function and maturation of tumor immune cells (19). So far, the overall impact of m^7^G-related regulators on the immunotherapeutic response in LUAD and its relationship with patient prognosis and treatment are still unclear.

With the advances in high-throughput sequencing technique, research on tumor genes is more in-depth, which can help to classify tumors to some content. There are many signatures that assess the prognosis of LUAD according to various subtypes (20–22). However, these signatures are still far from guiding precise treatment, which urgently requires a reliable signature. Here, two molecular subtypes of LUAD were constructed according to the gene expression of m^7^G-related regulators. We further evaluated the relation between survival, clinical characteristics, immune infiltration and molecular subtype. Then, a novel m^7^G score was established to quantify the m^7^G modification patterns, which was proven to be an independent predictor of LUAD prognosis. Moreover, the prognostic signature effectiveness was validated by the internal and external cohort (GSE31210). Furthermore, we elucidated whether this signature could provide reference for clinical immunotherapy and chemotherapy. In conclusion, this study not only provides a novel understanding of molecular subtype by m^7^G regulators, but also built a robust signature to estimate prognosis and guide individualized treatments in LUAD.

## Materials and methods

### Lung adenocarcinoma datasets

We obtained gene expression profile, clinical and somatic mutation data of LUAD from The Cancer Genome Atlas (TCGA) database.^1^ Four hundred ninety LUAD cases were included in the follow-up study after removing patients with survival time less than 30 days. We acquired the external verification cohort (GSE31210) from Gene Expression Omnibus (GEO) database.^2^ 226 cases were finally included after processing as TCGA data. 29 m^7^G regulators were extracted from previous report (23) and three m^7^G-related gene sets in MSigDB database^3^ (Supplementary Table 1).

### Landscape of genetic variation and identification molecular subtype

The expression of m^7^G regulators was extracted from TCGA-LUAD dataset. Then various methods were applied to depict the genetic variation of m^7^G regulators. The expression of m^7^G regulators was compared between tumor and normal groups. The mutation map was presented by using “maftools” package. Next, Cox analysis was used to filter genes correlated with LUAD prognosis (*P* < 0.05).

The “ConsensusClusterPlus” R package was employed to identify molecular subtype by consensus clustering of prognostic gene (parameters: reps = 50, pItem = 0.8, pFeature = 1, clusterAlg = “pam,” distance = “Pearson”) (24). Pam and Pearson distances were used as the clustering algorithm and distance measure, respectively. Furthermore, the sample’s distribution was characterized by principal component analysis (PCA). Moreover, we employed “survival” package to investigate the survival differences among subtypes. Besides, “heatmap” package was applied to explore the relation among molecular subtypes, expression of prognostic gene and clinicopathological features.

### Biological function analysis and immune infiltration profile estimation

We investigated the biological process of distinct molecular subtype by Gene Set Enrichment Analysis (GSEA). The “h.all.v7.5.1.symbols.gmt” gene set was obtained from MSigDB database. We applied the CIBERSORT algorithm (25) to assess the immune status among different molecular subtypes. In recent years, tumor mutational burden (TMB) was widely applied to measure the effectiveness of ICB therapy (26). TMB score was calculated by using the somatic mutation data of each patient and then we compared TMB score in different subtypes. In addition, patients were further separated into low and high TMB groups on the basis of the threshold value (27) (10 mutations/megabase) of TMB and then we compared the frequency of high TMB in different subtypes. GSE135222 immunotherapy cohort including 27 cases was obtained from GEO database, which used to verify immunotherapy efficacy of different subtypes.

### Screening of differentially expressed genes

We calculated the differentially expressed genes (DEGs) between molecular subtypes by using the Bioconductor “limma” package. The significance criteria were | log2FC| > 1 and false discovery rate (FDR) < 0.01. The upregulated and downregulated of DEGs were visualized by volcano map. The heatmap was also applied to show the distribution of DEGs in different subtypes.

### Construction and validation of the m^7^G related signature

The all patients (*n* = 490) were randomly separated into train and test cohort according to the ratio of 1:1 by using “caret” package. In train cohort (*n* = 245), univariate Cox analysis was applied to screen genes related to the survival (*P* < 0.01). Then, least absolute shrinkage and selection operator (LASSO) regression was employed to further reduce the overfitting genes. Finally, a m^7^G related signature was established by multivariate Cox analysis, and we also called it m^7^G score. The previously reported formula (28) was used to calculate m^7^G score: Σ(gene expression level × corresponding coefficient). Patients was separated into high and low m^7^G score groups according to median m^7^G score. Then the sample’s distribution was characterized by PCA. We applied “survival” package to investigate the survival differences between two groups. We also plotted the receiver operating characteristics (ROC) curve to estimate the accuracy of the m^7^G Related signature by using “timeROC” package. The test cohort (*n* = 245) and the entire cohort (*n* = 490) were employed to validate the signature power by using the same analyses. We further used GSE31210 dataset (*n* = 226) to verify the robustness of the signature.

### m^7^G related signature analysis and nomogram construction

Univariate and multivariate analysis were applied to demonstrate the independent prognosis of the m^7^G score. Then these results were visualized with the forest plots. We also constructed a nomogram by combining the age, gender, stage and m^7^G score for clinical practice. Additionally, calibration curve was applied to evaluate the predictive accuracy of the nomogram by using “rms” package.

### Analysis of immune infiltration and anti-cancer treatment

We applied different bioinformatics methods including XCELL, TIMER, QUANTISEQ, MCPCOUNTER, EPIC, CIBERSORT-ABS, CIBERSORT, and single-sample gene set enrichment analysis (ssGSEA) (29, 30) to study the relation between m^7^G score and immune score. Subsequently, we also compared the expression of immune checkpoints between two groups. Mutation maps were manifested by using “maftools” package in two groups. In recent years, in addition to immune checkpoints, tumor immune dysfunction and exclusion (TIDE) was also widely employed to assess the effectiveness of ICB therapy (31). The TMB score was calculated by using the somatic mutation data of each patient and the TIDE score was calculated in the TIDE website^4^ (*P* < 0.05). Moreover, the drug sensitivity of each group was estimated by “pRRophetic” package (32). The half-maximal inhibitory concentration (IC50) of drugs was compared through Wilcoxon rank test between different m^7^G score groups (*P* < 0.05).

### Statistical analysis

R software (version 4.1.0) was employed for all data analysis. Wilcoxon rank test was applied to compare m^7^G regulators expression between normal and LUAD groups. All above survival distribution was evaluated through survival analysis. The relation among molecular subtype, clinicopathological features and high TMB distribution was estimated by the chi-squared test. Immune infiltration, TMB and TIDE were also compared through Wilcoxon rank test. *P* < 0.05 was regarded as statistically significant.

## Results

### Genetic variation profile and m^7^G modification pattern

**FIGURE 1:**
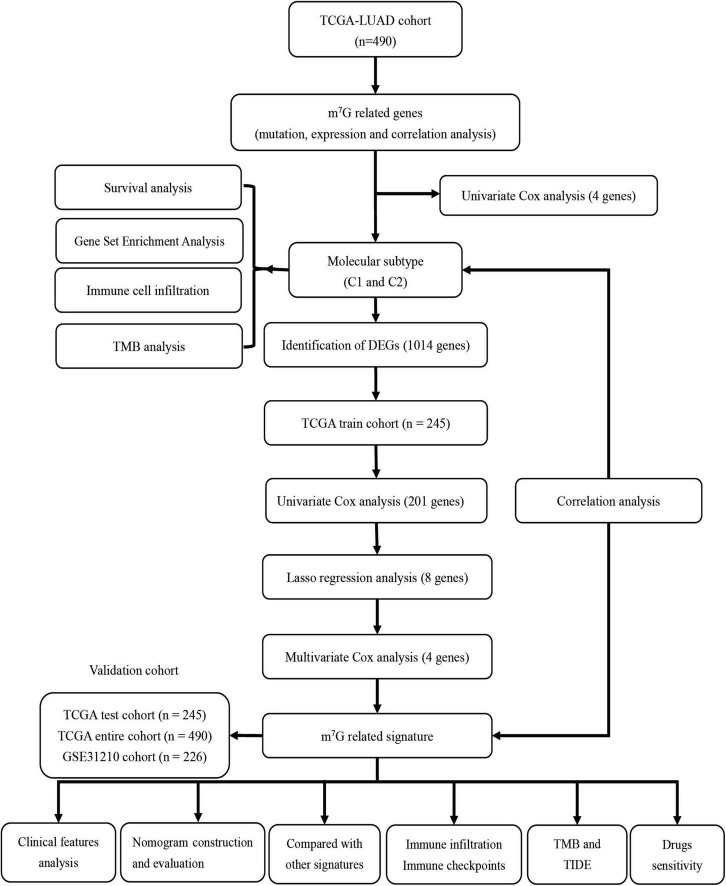
The flowchart of the overall study.

**FIGURE 2:**
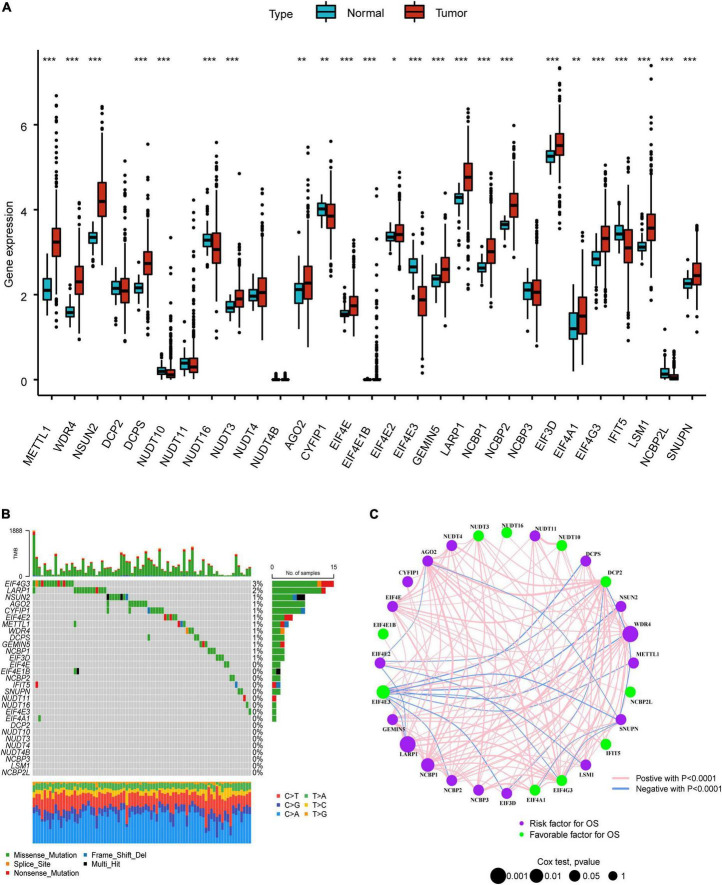
Landscape of genetic variation in LUAD. **(A)** The difference of m^7^G regulators expression level in normal and tumor group. **(B)** The mutation map of m^7^G regulators in LUAD. **(C)** The interplay and prognostic significance of m^7^G regulators. *P < 0.05; **P < 0.01; ***P < 0.001.

The overall research procedure is shown in Figure 1. Twenty-four regulators were manifested significant downregulation or overexpression in different groups according to *P*-value less than 0.05 (Figure 2A). The result of mutation map showed that *EIF4G3* had the highest mutation frequency followed by *LARP1* (Figure 2B). The correlation and prognostic significance of m^7^G regulators were presented in Figure 2C. Four genes including *EIF4E3*, *LARP1*, *WDR4*, and *NCBP1* were significantly associated with prognosis. m^7^G regulators were also showed a remarkable interaction, which was critical for the development of the different m^7^G modification patterns. The above-mentioned results suggested m^7^G regulators may relate to tumorigenesis and progression in LUAD.

**FIGURE 3:**
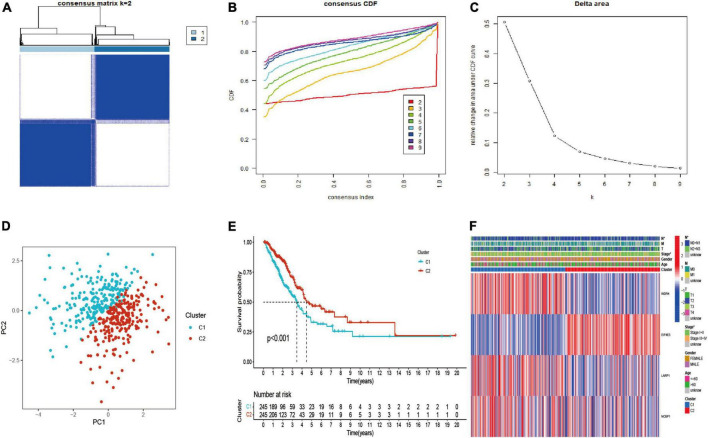
Identification, survival, and clinical characteristics analysis of molecular subtype. **(A–C)** The optimal value of consensus clustering. **(D)** Distribution of all patients. **(E)** Survival analysis in C1 and C2 subtypes. **(F)** Heatmap of prognostic gene and clinicopathological features between two subtypes. *P < 0.05.

The LUAD patients were classified into two molecular subtypes (C1 and C2) by using “ConsensusClusterPlus” package according to prognostic genes. The intergroup correlation was lowest and intragroup correlation was highest when *k* = 2 (Figure 3A). Cumulative distribution function (CDF) curve performed the highest partition efficiency when *k* = 2 (Figures 3B,C). Taken together, two molecular subtypes were established according to the m^7^G modification pattern, including 245 patients of C1 and 245 patients of C2. The PCA analysis also demonstrated that the two subtypes could be completely distinguished (Figure 3D). The result of Kaplan–Meier analysis showed distinct survival outcome between two subtypes (*P* < 0.001) (Figure 3E), suggesting that C1 subtype had worse prognosis than C2. Subsequently, the clinical features and gene expression were compared, then we found patients in C1 subtype had poorer tumor stage than C2. *EIF4E3* was upregulated in C2 subtype, while *LARP1*, *WDR4*, and *NCBP1* were upregulated in C1 subtype (Figure 3F).

### Analysis of biological functional and immune infiltration

**FIGURE 4:**
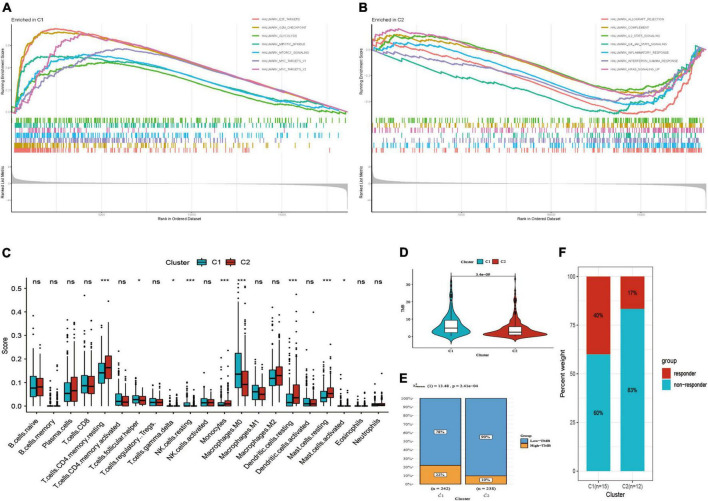
Functional pathways, immune infiltration and TMB analysis. **(A)** Functional pathways in C1 subtype. **(B)** Functional pathways in C2 subtype. **(C)** Comparison of immune score. **(D)** Comparison of TMB score. **(E)** Distribution of low and high TMB between two subtypes. **(F)** Distribution of response to immunotherapy between two subtypes in GSE135222. *P < 0.05; ***P < 0.001; ns: not significant.

The results of GSEA presented diverse functional pathways between two subtypes. Functional analysis showed E2F_targets, G2M_checkpoint, glycolysis, MITOTIC spindle, MTORC1_signaling, MYC_targets_V1, MYC_targets_V2 were significantly enriched in C1 subtype (Figure 4A). While, there were significantly different pathways were enriched in C2 subtype, such as allograft rejection, complement, inflammatory response, interferon gamma response, IL2_STAT5_signaling, IL6_JAK_STAT3_signaling (Figure 4B).

Subsequently, we found that two subtypes had markedly different immune infiltration patterns (Figure 4C). The result of CIBERSORT algorithm showed the expression level of T cells follicular helper, resting NK cells, M0 macrophages, activated mast cells were high in C1 subtype, while resting CD4 memory T cells, T cells gamma delta, monocytes, resting dendritic cells, resting mast cells are high in C2 subtype. These suggested that two subtypes may have different immunotherapeutic response, so we further compared TMB score in two subtypes. Then we observed that C1 had higher TMB score as well as the proportion of high-TMB compared to C2 subtype (22 vs. 10%) (Figures 4D,E). GSE135222 immunotherapy cohort was divided into two subtypes by using the same method mentioned above, including 15 cases in C1 subtype and 12 cases in C2 subtype. The results presented that the proportion of response to immunotherapy was higher in C1 subtype than C2 subtype (40 vs. 17%) (Figure 4F).

### Screening differentially expressed genes between m^7^G subtypes and construction of m^7^G related signature

**FIGURE 5:**
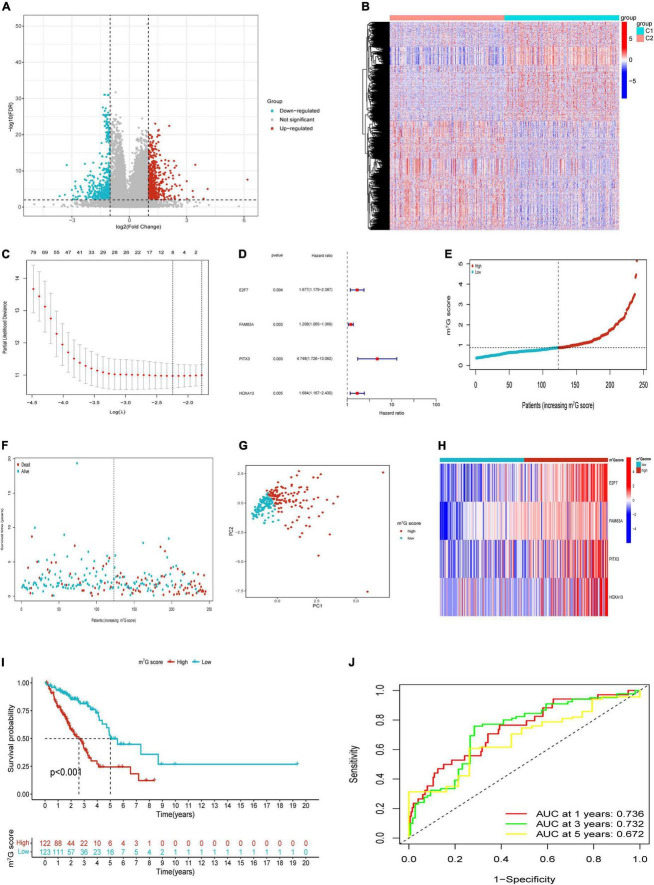
Construction of m^7^G related signature based on TCGA train cohort. **(A)** Volcano plot of DEGs. **(B)** Heatmap of DEGs. **(C)** Eight genes through Lasso regression analysis. **(D)** Four key genes through multivariate Cox regression. **(E,F)** Distribution of m^7^G score and survival state. **(G)** Distribution of patients according to m^7^G-related signature. **(H)** Heatmap of four genes expression between high and low m^7^G score groups. **(I)** Survival analysis in two groups. **(J)** AUC for predicting 1-, 3-, 5-years survival rates.

Based on “limma” package, we identified 1,014 DEGs between m^7^G Subtypes, including 534 upregulated genes and 480 downregulated genes. Then the significant DEGs were visualized with volcano map (Figure 5A). The expression profiles of DEGs between C1 and C2 subtype were visualized with heatmap (Figure 5B). We utilized the train cohort to establish m^7^G related prognostic signature (*n* = 245). First, 201 genes correlated with patient prognosis were found by univariate analysis (Supplementary Table 2). Then we further applied LASSO regression to filter eight genes for the subsequent multivariate analysis (Figure 5C). Finally, four key genes including *E2F7*, *FAM83A*, *PITX3*, and *HOXA13* were identified by using multivariate Cox regression (Figure 5D). The m^7^G score was calculated with the following formula: 0.5171 *× E2F7* (mRNA level) + 0.1888 *× FAM83A* (mRNA level) + 1.5576 *× PITX3* (mRNA level) + 0.5210 *× HOXA13* (mRNA level). Then we split patients into high and low m^7^G score groups according to approach mentioned above (Figures 5E–G). The relative expressions of *E2F7*, *FAM83A*, *PITX3*, and *HOXA13* in two groups were presented in Figure 5H. Patients had significant poor survival in high m^7^G score group (Figure 5I). The areas under the curves (AUC) for predicting survival rates at 1-, 3-, and 5-year were 0.736, 0.732, and 0.672, respectively (Figure 5J).

### Verification of m^7^G related signature and analysis of survival in different clinical subgroups

**FIGURE 6:**
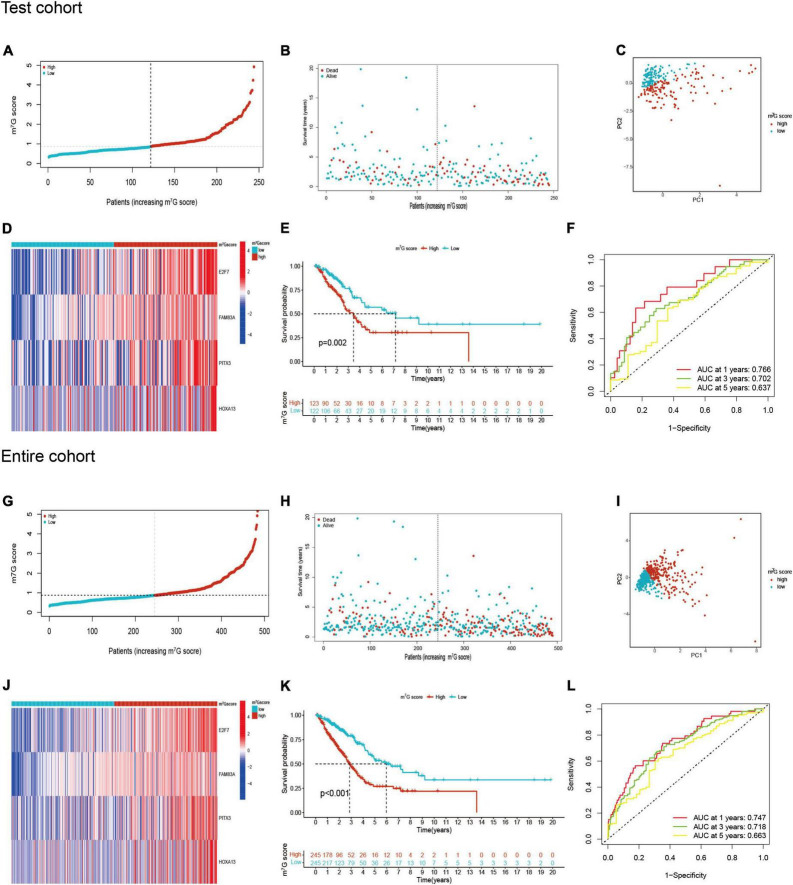
m^7^G related signature validation in TCGA test cohort and TCGA entire cohort. **(A,B)** Distribution of m^7^G score and survival state in TCGA test cohort. **(C)** Distribution of patients in TCGA test cohort. **(D)** Four genes expression between two groups in TCGA test cohort. **(E)** Survival analysis between two groups in TCGA test cohort. **(F)** AUC for predicting 1-, 3-, 5-years survival rates in TCGA test cohort. **(G–L)** The results of validation in TCGA entire cohort.

**FIGURE 7:**
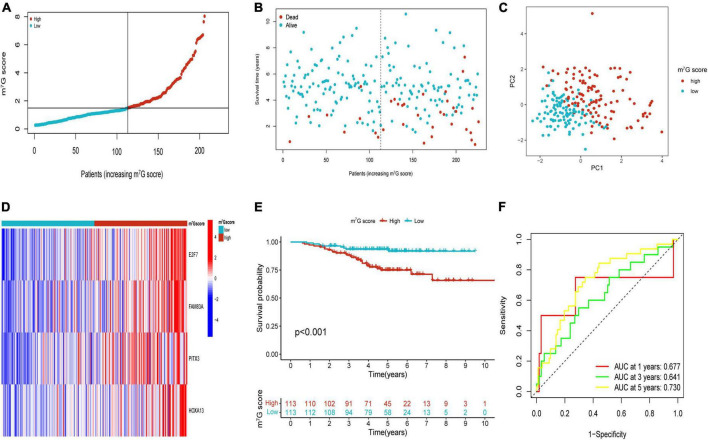
Validation of m^7^G related signature in GSE31210. **(A,B)** The distribution of m^7^G score and survival state. **(C)** Distribution of patients according to m^7^G-related signature. **(D)** Heatmap of four genes expression between two groups. **(E)** Survival analysis between two groups. **(F)** AUC for predicting 1-, 3-, 5-years survival rates.

Patients in the internal verification cohort (test and entire cohort) and the external verification cohort (GSE31210) were categorized into two groups on the basis of the same risk formula in train cohort. Patients of test cohort were classified into two groups (Figures 6A–C), which were consistent with the result of the train cohort. The heatmap showed the expression profile of four key gene were apparently different between two groups (Figure 6D). The Kaplan–Meier curve also indicated that two groups had distinct survival (Figure 6E). The area of AUC verified that the signature was a great indicator for assessing prognosis in LUAD (Figure 6F). The similar results were acquired in the entire cohort and GSE31210 cohort (Figures 6G–L, 7A–F). On the basis of Kaplan–Meier analysis of entire TCGA cohort, we also found that patients presented lower survival rate in high m^7^G score group among different clinical subgroups compared to low m^7^G score group (Supplementary Figure 1).

### Evaluation of association between m^7^G score, clinicopathological features, and molecular subtype

**FIGURE 8:**
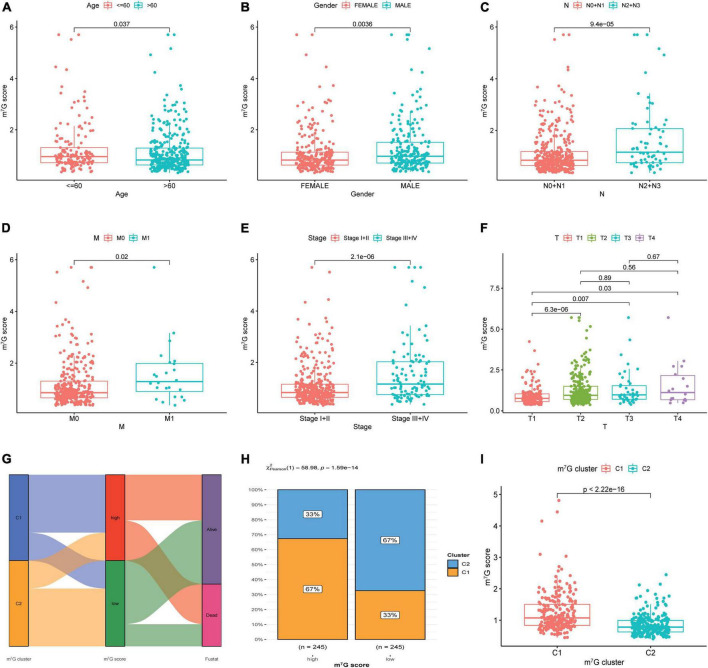
Correlation among clinical features, molecular subtype and m^7^G score. **(A–F)** Correlation between clinical features and m^7^G score. **(G)** Relation between m^7^G score, m^7^G subtype and survival state by using Sankey diagram. **(H)** Distribution of m^7^G subtype between high and low m^7^G score groups. **(I)** Comparison of m^7^G score between C1 and C2 subtypes.

The results showed strikingly distinct of m^7^G score in age, gender, N-stage, M-stage, clinical stage and T-stage (Figures 8A–F) (*P* < 0.05). We also investigated the relation between m^7^G score, m^7^G subtype and survival state by using Sankey diagram (Figure 8G). We found that C2 subtype has a strong correlation with low m^7^G score, while C1 subtype has a strong correlation with high m^7^G score. Moreover, the majority of patients with low m^7^G score were alive, which was consistent with preceding survival analysis. Furthermore, stacked bar chart also presented C1 subtype has a strong correlation with high m^7^G score (Figure 8H). Similarly, the m^7^G score was higher in C1 subtype than that in C2 subtype (Figure 8I).

### Construction of nomogram and comparison of prognostic signatures

**FIGURE 9:**
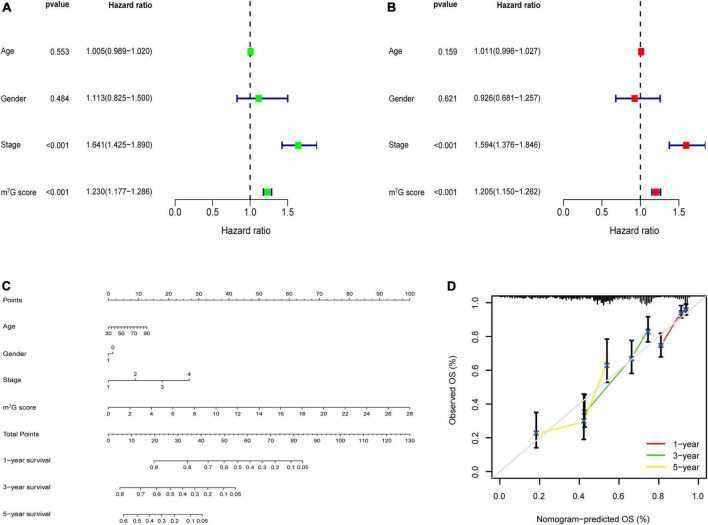
m^7^G related signature analysis and nomogram construction. **(A)** Univariate analysis of m^7^G score and clinical factors. **(B)** Multivariate analysis of m^7^G score and clinical factors. **(C)** Establishment of nomogram. **(D)** Calibration curve of the nomogram.

Univariate analysis identified that m^7^G score was related to poor prognosis in LUAD (Figure 9A). Moreover, m^7^Gs core was still an independent prognostic indicator after using multivariate analysis (Figure 9B) (*P* < 0.001). Subsequently, we used m^7^G score and other clinical factors to establish a nomogram (Figure 9C). Calibration curve demonstrated that 1, 3, 5-year predicted survival rates matched the veritable condition (Figure 9D). These evidences revealed that the m^7^G score could potentially assist clinical practice to evaluate the prognosis of LUAD patients.

**FIGURE 10:**
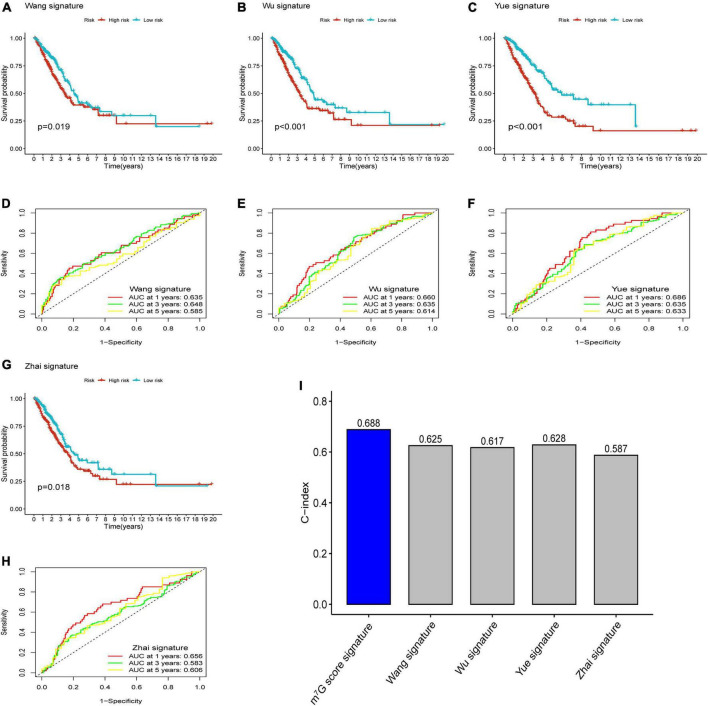
Comparison of prognostic signatures. **(A–C,G)** Survival analysis of 5-gene signature (Wang), 4-gene signature (Wu), 3-gene signature (Yue) and 5-gene signature (Zhai). **(D–F,H)** AUC of 5-gene signature (Wang), 4-gene signature (Wu), 3-gene signature (Yue), and 5-gene signature (Zhai) for predicting 1-, 3-, 5-years survival rates. **(I)** C-index of each signature.

After reviewing previous researches, we further compared the m^7^G related signature with other prognostic models, including 5-gene signature (Wang) (33), 4-gene signature (Wu) (34), 3-gene signature (Yue) (35), and 5-gene signature (Zhai) (36). In order to ensure comparability among models, the risk score of each LUAD sample in entire TCGA cohort was calculated with the same formula according to corresponding genes in four signatures, and then patients were categorized into two groups based on same cut-off value (37). The results of survival analysis showed significant difference in four models (Figures 10A–C,G) (*P* < 0.05). However, all AUC at 1-, 3-, and 5-years in four models were lower than that corresponding AUC of our prognostic signature (Figures 10D–F,H). Furthermore, we conducted “survcomp” package to calculate the concordance index (C-index) of each signature. The C-index was highest in our prognostic signature (Figure 10I). Therefore, our signature was more efficient to estimate prognosis in LUAD.

### Investigation of immune microenvironment and anti-cancer therapy

**FIGURE 11:**
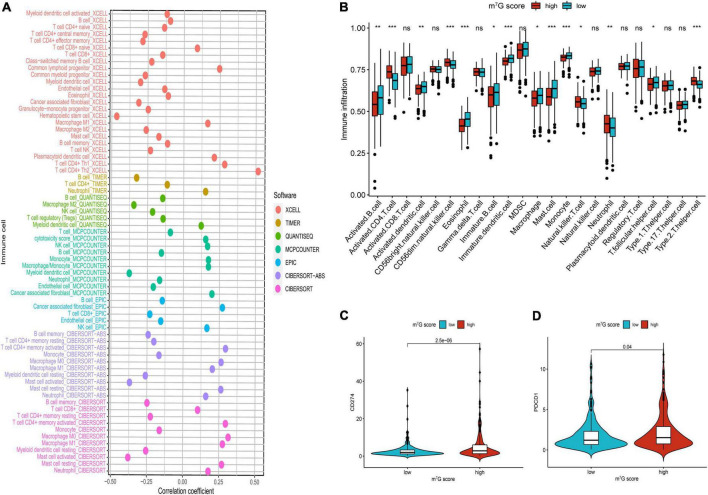
Comparison of immune infiltration and immune checkpoints between high and low m^7^G score groups. **(A)** Correlation between immune cell and m^7^G score. On the right side of the correlation coefficient = 0 indicates a positive correlation with m^7^G score. **(B)** Immune infiltration analysis by ssGSEA. **(C)** Comparison of CD274 (PD-L1). **(D)** Comparison of PDCD1 (PD-1). *P < 0.05; **P < 0.01; ***P < 0.001.

**FIGURE 12:**
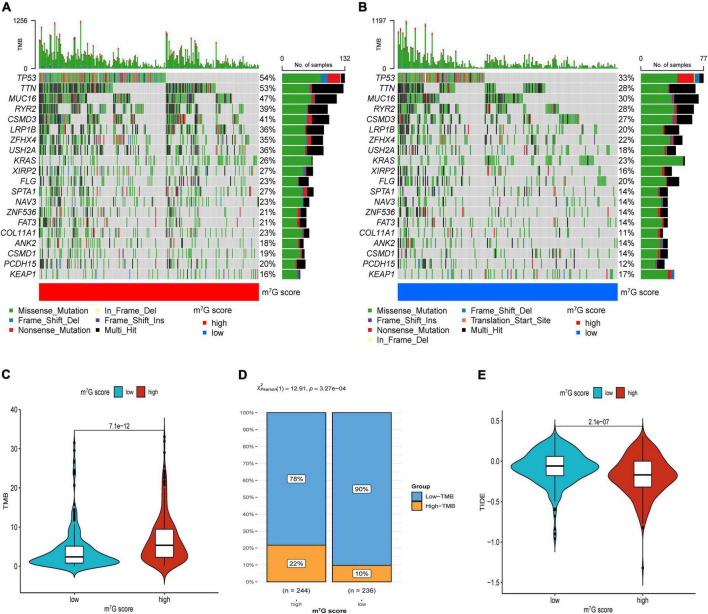
Immunotherapeutic response evaluation in high and low m^7^G score groups. **(A)** Mutation plot in high m^7^G score group. **(B)** Mutation plot in low m^7^G score group. **(C)** Results of TMB between two groups. **(D)** Distribution of low and high TMB between two subtypes. **(E)** Results of TIDE between two groups.

Firstly, the results of bubble plot exhibited that CD8+ T cell, common lymphoid progenitor, plasmacytoid dendritic cell, macrophage M1/M0, CD4+ Th1/Th2 cell, neutrophil, cytotoxicity score, NK cell, cancer associated fibroblast, monocyte, Myeloid dendritic cell, and mast cell resting were positively correlated with m^7^G score (Figure 11A). The ssGSEA displayed that activated CD4 T cell, CD56dim natural killer cell, natural killer T cell, neutrophil, Type-2 T helper cell were more active in high m^7^G score group (Figure 11B) (*P* < 0.05). Subsequently, the level of CD274 (PD-L1) and PDCD1 (PD-1) were presented upregulated in high m^7^G score group (Figures 11C,D) (*P* < 0.05). Moreover, we compared somatic mutations in two risk groups. The results of mutation map showed remarkably high mutational rate in high m^7^G score group. *TP53* was the highest mutational gene in both groups (Figures 12A,B). We also found that high m^7^G score group was related to higher TMB score (Figures 12C,D) (*P* < 0.05). Compared to low m^7^G score group, high m^7^G score group had strikingly lower TIDE score (Figure 12E) (*P* < 0.05).

**FIGURE 13:**
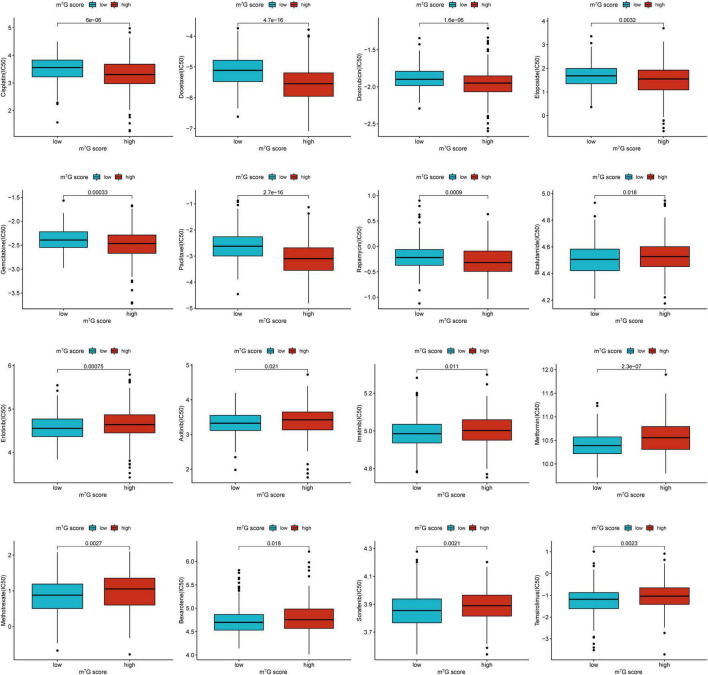
Comparison of drug sensitivity between low and high m^7^G score groups.

We further investigated common drug sensitivity in two groups (Figure 13). Patients presented lower IC50 of Cisplatin, Docetaxel, Doxorubicin, Etoposide, Gemcitabine, Paclitaxel, and Rapamycin in high m^7^G score group, representing these drugs were more effective for high m^7^G score group. Meanwhile, IC50 of Bicalutamide, Erlotinib, Axitinib, Imatinib, Metformin, Methotrexate, Bexarotene, Sorafenib, and Temsirolimus were lower in low m^7^G score group, representing these drugs were more effective for low m^7^G score group.

## Discussion

Recently, the role of m^7^G in tumors has begun to receive increasing attention. However, there are no reports on studying the molecular subtype correlated with m^7^G and the implications of m^7^G related signature on the prognosis and immunotherapy in LUAD. Therefore, we expect to discover more tumor phenotypes through this classification, which could be used to evaluate the prognosis of LUAD patients. Here, we first extracted twenty-nine m^7^G related regulators expression profiles from TCGA, four of which were demonstrated to have prognostic value. Then, two novel molecular subtypes were identified according to theses prognostic genes. Results showed that patients in C1 subtype had more poor survival outcomes and advanced tumor stages compared to C2 subtype through survival analysis and clinicopathological features comparison, indicating m^7^G regulators correlated with prognosis and progression of LUAD. And the two subtypes presented markedly different molecular features. Compared with C2 subtype, m^7^G regulators were more activated in C1 subtype, including *LARP1*, *WDR4*, and *NCBP1*, while only *EIF4E3* was activated in C2 subtype. Xu et al. (38) demonstrated that the expression level of *LARP1* was upregulated in NSCLC, which positively related to poor prognosis and progression of cancer. A study of *WDR4* uncovered that knockdown of *WDR4* could restrain the aggressiveness of NSCLC cells, demonstrating that *WDR4* may have tumorigenic function in lung cancer (18). *NCBP1* was significantly overexpressed in LUAD, combined with CUL4B, which promoted the proliferation, migration and invasion of tumor cells (39). It was reported that compared with patients with lower expression of EIF4E3, patients with high expression of *EIF4E3* had markedly better survival rates in various cancers, including LUAD (40). These results are consistent with our study, indicating LUAD patients with C1 subtype have poor prognosis compared to C2 subtype. Furthermore, we investigated the possible functional mechanisms in both two subtypes by using GSEA analysis. Interestingly, the functional pathways enriched in C1 subtype were mainly cell proliferation-related pathways, which may indicate advanced clinicopathological staging, adverse survival outcomes and aggressive tumor subtypes. This evidence also suggested the worse prognosis of C1 subtype. Immune cells as an important part of the tumor microenvironment intensively relate to the response to immunotherapy (41). Recently studies showed that RNA modifications were correlated with the differentiation of immune cells in the tumor microenvironment (42). As one of the RNA modifications, m^7^G also influenced immune cells in the tumor microenvironment. Chen et al. (43) quantified the tumor-infiltrating lymphocytes and found that CD4+ T exhaustion and Tregs decline after knockout of m^7^G regulators. Besides, Devarkar et al. (44) presented that m^7^G was involved in innate immunity mediated by RIG-I. Therefore, the immune score was applied to characterize immune microenvironmental landscapes in two subtypes. Then we found that follicular helper T cells, resting NK cells and activated mast cells were increased in C1 subtype. It was reported that follicular helper T cells could recruit CD8+ T cells to enhance antitumor immune response (45). The resting NK cells could secrete various cytokines to kill target cells, for example tumor cells (46). The TLR4 activation by mast cells resulted in the secretion of CXCL10, which could recruit effector T cells to influence antitumor immune response (47). These evidences indicated that C1 subtype with activated m^7^G regulators may more sensitive to immunotherapy compared to C2 subtype. Increasing investigations suggested that TMB is a biomarker of response to immunotherapy and is positively relate to the effectiveness of ICB in various cancers, including NSCLC (48). In this study, the TMB score in patients with C1 subtype was distinctly higher compared with C2 subtype. After dividing TMB into high and low groups, more percentage of high TMB was observed in patients with C1 subtype compared with C2 subtype. Consequently, patients with C1 subtype may present better immunotherapeutic response than C2 subtype. Besides, by using an immunotherapy cohort of lung cancer, we also demonstrated that patients with C1 subtype had better immunotherapy efficacy than C2 subtype.

Considering the individual heterogeneity of m^7^G modification, we utilized a novel m^7^G score to quantify the m^7^G modification patterns in LUAD. In the TCGA train cohort, we identified four key genes (*E2F7*, *FAM83A*, *HOXA13*, and *PITX3*), and then calculated the m^7^G score through the previously mentioned algorithm. After separating patients into high and low m^7^G score groups, we found that the four genes were overexpressed in the high m^7^G score group. It was reported that overexpression of *E2F7* correlated with poor prognosis and microRNA-935 could inhibit tumor metastasis and invasion by targeted suppression the level of *E2F7* in NSCLC (49). Wang et al. (50) found that activating the expression of *E2F7* expression by targeting microRNA-140-3p could promote the progression of LUAD. Studies presented that *FAM83A* was significantly related to TMB and DNA damage response pathways in NCSLC (51, 52), indicating that it may play an important part in tumor progression and immunotherapy. Hu et al. (53) demonstrated that the expression of *FAM83A* regulated the proliferation and invasiveness of NSCLC through PI3K/Akt/mTOR pathway. Investigations showed *HOXA13*, as a nuclear transcription factor, was related to tumor cells proliferation and differentiation, which could accelerate tumor aggressive characteristics through disturbing P53 and Wnt/β-catenin signaling pathways in NSCLC (54). One research reported that *HOXA13* was markedly upregulated and strongly correlated with tumorigenesis and progression in LUAD (55). Some studies demonstrated that *PITX3* as a transcription factor was involved in many tumors (56, 57). Zhang et al. (58) presented that high expression of *PITX3* was strongly associated with the poor prognosis in LUAD. According to these evidences, we indicated that patients with high m^7^G score in which these four genes were activated, had poor survival. Also, survival analysis demonstrated that the high m^7^G score group presented worse survival outcomes. ROC curves further showed the great efficacy of m^7^G score to predict survival rate. And, the TCGA test cohort, entire cohort and GSE31210 cohort were applied to validate the accuracy and reliability of the m^7^G related signature. Similar results were acquired from all validation cohorts, demonstrating that the prognostic signature may be a robust biomarker to evaluate the prognosis in LUAD. Patients with high m^7^G score also presented remarkably poor survival condition among different clinical subgroups. After analyzing the association between m^7^G score and clinicopathological parameters, we observed that m^7^G score was significantly high in N2 + N3, M1, Stage III + IV and T2-T4, suggesting high m^7^G score is associate with cancer progression. The characterization of m^7^G modification patterns showed C2 subtype had lower m^7^G score compared with C1 subtype. And, the high m^7^G score correlated with poor survival and cancer progression was consistent with characteristics of C1 subtype. Univariate and multivariate analysis presented that the m^7^G score was an independent prognosis predictor of LUAD patients. Subsequently, the nomogram also presented high accuracy in predicting survival rate of 1, 3, 5-years. In recent years, many signatures were built to predict the prognosis of LUAD patients. Furthermore, we presented that the AUC area and C-index of our signature were higher than the other four public prognostic signatures, suggesting our signature have better performance in predicting clinical prognosis in LUAD patients.

Currently, although the immunotherapy of lung cancer has got great progress, how to choose the appropriate therapeutic regime for patients is still a clinical challenge. Besides, a part of patients did not obtain effective benefits from immunotherapy (59), and even some patients will undergo obvious side effects during therapy (60). Therefore, it is critical to explore a novel method to guide individualized and precise treatment in LUAD patients. The results of various evaluation methods of immune cell infiltration showed distinct activation of immune cells in both groups, which were similar to molecular subtypes. We speculated there was different immunotherapeutic response in two groups, so we further investigated the association between m^7^G score groups, immune checkpoint, TMB and TIDE. The immune checkpoints are also an integral part of the immune system and participate in regulating immune escape (61). In recent years, immunotherapy targeting immune checkpoints has obtained huge clinical therapeutic results, especially anti-PD-1/PD-L1 antibody (62). In the study, we observed that patients with high m^7^G score had upregulated PD-1/PD-L1, indicating that these patients may be more sensitive to ICB than low m^7^G score. Subsequently, compared with the low m^7^G score group, the high m^7^G score group had markedly higher TMB which was consistent with the C1 subtype. In addition, we found that compared with the patients with low m^7^G score, patients with high m^7^G score had more percentage of *TP53* mutation. Studies showed that *TP53* mutation was remarkably related to high PD-L1 expression and patients with *TP53* mutation could acquire benefits from ICB therapy in LUAD (63, 64). Investigations presented TIDE was an accurate biomarker used to predict the immunotherapeutic effects of NSCLC, which was negatively associated with the efficacy of ICB (31). Meanwhile, recent studies have reported the clinical application of TIDE in predicting and evaluating immunotherapeutic response (65, 66). In our study, compared with patients with low m^7^G score patients with high m^7^G score had lower TIDE score, suggesting patients in the high m^7^G score group may obtain clinical benefits from immunotherapy. Integration analysis of the m^7^G score, immune cell infiltration, immune checkpoint, TMB, and TIDE indicated that the signature is a potential biomarker to assess immunotherapeutic response and tailor individualized treatment for LUAD patients.

Chemotherapy is a classic treatment for lung cancer, but patients have different response rates to chemotherapy drugs. Selecting an appropriate chemotherapy regimen is helpful to improve the prognosis and reduce the economic burden of patients. Our study revealed that common drugs including Cisplatin, Docetaxel, Doxorubicin, Etoposide, Gemcitabine, Paclitaxel, and Rapamycin were suitable for patients with high m^7^G score, while Axitinib, Bexarotene, Bicalutamide, Erlotinib, Imatinib, Metformin, Methotrexate, Sorafenib, and Temsirolimus were more appropriate for patients with low m^7^G score.

Our research may assist in judging the prognosis in LUAD, but there are also some limitations. First, the research is a retrospective study according to the data from TCGA and GEO datasets, so it’s crucial to collect prospective clinical data to further verify the signature. Second, the potential functional mechanisms of m^7^G score are not fully verified, so these need to be further verified by experiments at the molecular level *in vivo* and *in vitro*. Finally, the drug response in patients is based on methodological prediction, so clinical trials need to be implemented in the future.

## Conclusion

In summary, we identified two novel molecular subtypes of LUAD according to m^7^G regulators. The survival, immune infiltration, and TMB are significantly different in two subtypes. The m^7^G related signature to quantify the heterogeneity of the two subtypes was constructed. The signature can be employed to predict prognosis in LUAD, then the internal and external cohort were applied to verify the prognostic value. And the signature was elucidated be helpful to guide immunotherapy and chemotherapy. Therefore, this research provides a new direction for improving prognosis and current anti-cancer strategies in LUAD.

## Data availability statement

The datasets presented in this study can be found in online repositories. The names of the repository/repositories and accession number(s) can be found in the article/Supplementary material.

## Ethics statement

Ethical review and approval was not required for the study on human participants in accordance with the local legislation and institutional requirements. Written informed consent for participation was not required for this study in accordance with the national legislation and the institutional requirements.

## Author contributions

ZL and XY conceived and designed the study. ZL and WW performed the data analysis. ZL wrote the manuscript. WW and JW participated in collecting the data and helped draft the manuscript. XY and WW prepared and edited the manuscript. All authors reviewed and approved the manuscript.

## Footnotes

1
https://portal.gdc.cancer.gov/

2
https://www.ncbi.nlm.nih.gov/geo/

3
http://www.gsea-msigdb.org/gsea/index.jsp

4
http://tide.dfci.harvard.edu/

## Supplementary material

Supplementary Figure 1 Survival analysis among different clinical subgroups.

Supplementary Table 1 Three m^7^G-related gene sets.

Supplementary Table 2 201 prognostic genes through univariate Cox analysis.

## Abbreviations

LUAD: lung adenocarcinoma
m^7^G: N7-methylguanosine
TCGA: The Cancer Genome Atlas
TMB: tumor mutational burden
DEGs: differentially expressed genes
NSCLC: non-small cell lung cancer
ICB: immune checkpoint blockade
GEO: Gene Expression Omnibus
PCA: principal component analysis
GSEA: Gene Set Enrichment Analysis
FDR: false discovery rate
LASSO: least absolute shrinkage and selection operator
ROC: receiver operating characteristics
ssGSEA: single-sample gene set enrichment analysis
TIDE: tumor immune dysfunction and exclusion
IC50: half-maximal inhibitory concentration
CDF: cumulative distribution function
AUC: areas under the curves
C-index: concordance index.
