# Supplementary figures and images for: Identification of N7-methylguanosine related signature for prognosis and immunotherapy efficacy prediction in lung adenocarcinoma
Source: Front Med (Lausanne). 2022 Aug 24;9:962972. doi: 10.3389/fmed.2022.962972 (PMC9449120; doi:10.3389/fmed.2022.962972)

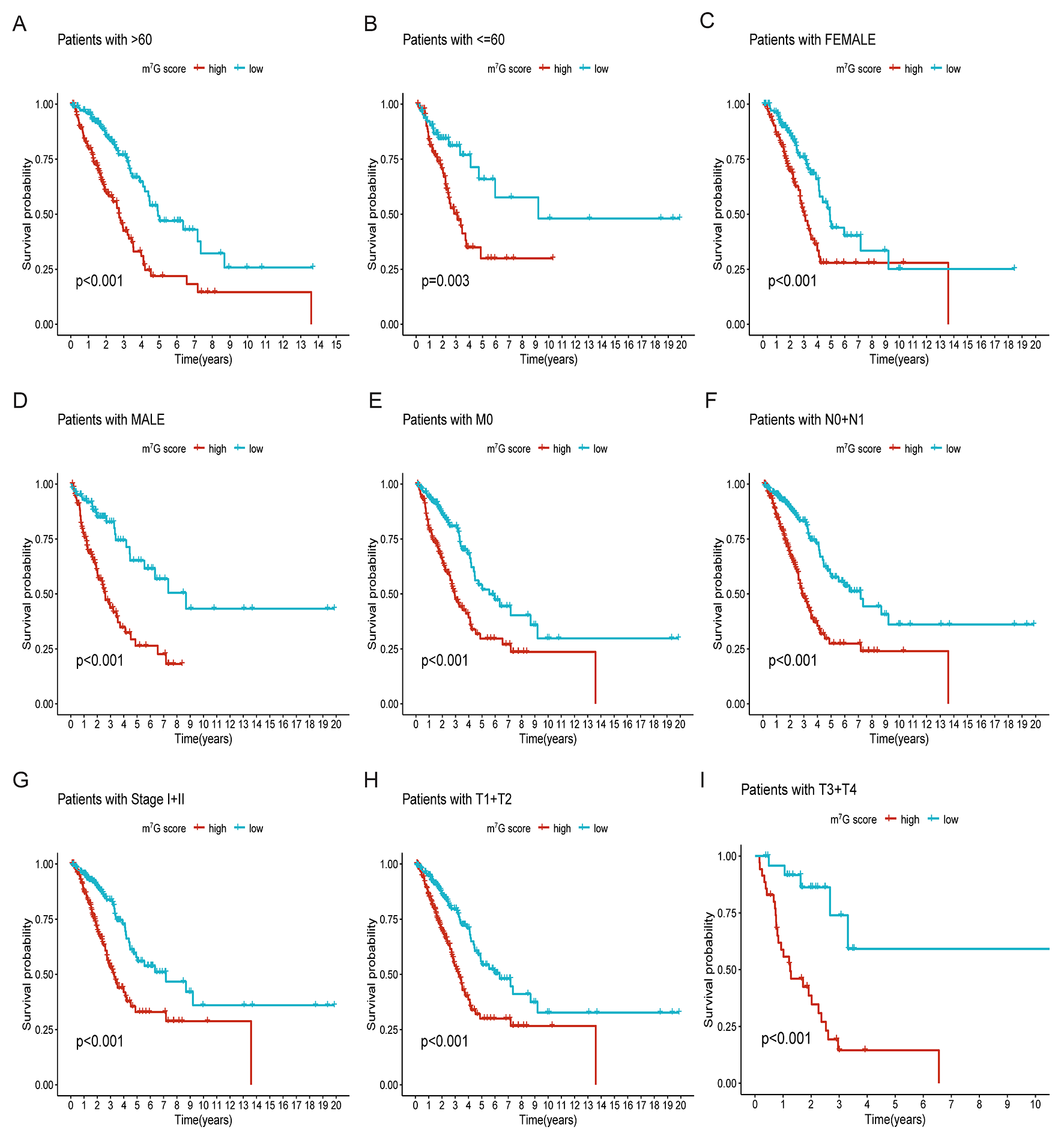

Supplement: Supplementary Figure 1 — Survival analysis among different clinical subgroups. [file Image_1.TIF]
